# Protein folding in HP model on hexagonal lattices with diagonals

**DOI:** 10.1186/1471-2105-15-S2-S7

**Published:** 2014-01-24

**Authors:** Dipan Lal Shaw, ASM Shohidull Islam, M Sohel Rahman, Masud Hasan

**Affiliations:** 1 AℓEDA Group, CSE, BUET, Bangladesh; 2 Department of CSE, BUET, Dhaka 1000, Bangladesh; 3 Department of CSE, BRAC University, Dhaka, Bangladesh; 4 Department of Computer Science, College of Computer Science and Engineering, Taibah University, Madina Munawwarah, Saudi Arabia

**Keywords:** bioinformatics, approximation algorithms, protein folding, lattice model, HP model, hexagonal lattice

## Abstract

Three dimensional structure prediction of a protein from its amino acid sequence, known as protein folding, is one of the most studied computational problem in bioinformatics and computational biology. Since, this is a hard problem, a number of simplified models have been proposed in literature to capture the essential properties of this problem. In this paper we introduce the hexagonal lattices with diagonals to handle the protein folding problem considering the well researched HP model. We give two approximation algorithms for protein folding on this lattice. Our first algorithm is a $\frac{5}{3}$-approximation algorithm, which is based on the strategy of partitioning the entire protein sequence into two pieces. Our next algorithm is also based on partitioning approaches and improves upon the first algorithm.

## Introduction

Protein folding is one of the most studied computational problems in bioinformatics. Many approximation solutions for this problem are given in the literature by using simplified, abstract models. There exist a variety of models attempting to simplify the problem by abstracting only the "essential physical properties" of real proteins. A lattice model for folding amino acids is represented by connected beads in two dimensional lattices or three dimensional cubic lattices and considers a simplified energy function. We can categorize the lattice models into two different classes: Simplified Lattice Models (e.g. [1]) and Realistic Lattice Models [2]. One of the widely used simplified lattice model is the HP model which was first introduced by Dill [1]. In HP model, there are only two types of beads: H represents a hydrophobic or non-polar bead and P represents a polar or hydrophilic one. The main force in the folding process is the hydrophobic-hydrophobic force, i.e., H-H contacts. For optimal embedding, our main goal is to maximize the H-H contacts.

The protein folding problem in HP model is NP-hard [3]. So a number of approximation algorithms have been developed for the HP model on different lattice structures. Hart and Istrail gave the first 4-approximation algorithm for the problem on the 2D square lattice [4]. Later on, Newman [5] improved the approximation ratio to 3 considering the conformation as a folded loop. A $\frac{8}{3}$-approximation algorithm for the problem on the 3D square lattice was given by Hart and Istrail [4]. In [6], the authors introduced square lattice with diagonals and presented algorithms that give an approximation ratio of $\frac{26}{15}$ for the two-dimensional and $\frac{8}{5}$ for the three-dimensional lattice. Later, Newman and Ruhl improved this based on different geometric ideas; they achieved an improved approximation ratio of 0.37501 [7]. To remove the parity problem of the square and cubic lattices Agarwala et al. first proposed the triangular lattice [8]. There, they gave a $\frac{11}{6}$ approximation algorithm. For a more generalized version, namely, the 3D FCC lattice, Agarwala et al. [8] gave an approximation algorithm having an approximation ratio of $\frac{5}{3}$. To alleviate the problem of sharp turns, Jiang and Zhu introduced the hexagonal lattice model and gave an approximation algorithm having approximation ratio of 6 [9]. A linear time approximation algorithm for protein folding in the HP side chain model on extended cubic lattice having an approximation ratio of 0.84 was presented by Heun [10].

A number of heuristic and meta-heuristic techniques have also been applied to tackle the protein folding problem in the literature. A genetic algorithm for the protein folding problem in HP model in 2D square lattice was proposed in [11]. In [12,13], a hybrid genetic algorithm was presented for HP model in 2D triangular lattice and 3D FCC lattice. The authors in [14] first proposed the *pull move set *for rectangular lattices, which is used in the HP model under a variety of local search methods. They also showed the completeness and reversibility of the pull move set for rectangular grid lattices. In [15], the authors extended the idea of the *pull move set *in the local search approach for finding an optimal embedding in 2D triangular grid and the FCC lattice in 3D.

**Figure 1:**
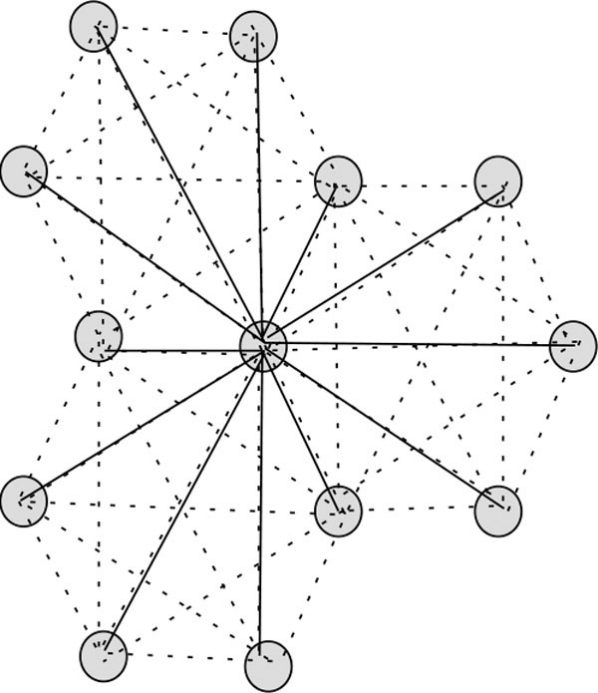
**Hexagonal lattice with diagonal**. In this figure, hexagonal lattice with diagonal is illustrated.

In this paper, we introduce the hexagonal lattices with diagonals for protein folding. The motivation for introducing hexagonal lattice comes from the secondary structure of a protein as follows. The secondary structure of a protein suggests that, in real protein folding, sharp turn does not occur frequently. Hexagonal model alleviates this sharp turn problem [9]. On the other hand, in the square lattice HP model there is a serious shortcoming, namely, the *parity problem *as follows. Due to a grid structure in a square lattice, contact can be established between two hydrophobic atoms only if they both are either on even positions or on odd positions of the sequence. To address this *parity problem*, we came up with the idea of this new lattice model, i.e., hexagonal lattice model with diagonals. In this model contacts may exist through diagonals (see Figure 1). Notably, these issues have also been partially alleviated in square lattice with diagonals and triangular lattice. To this end, our new model opens a new avenue for further research for this long standing problem. We present two novel approximation algorithms for protein folding on this lattice. Our first algorithm is a $\frac{5}{3}$-approximation algorithm for *k *> 10 where *k *is the number of sequences of H's in the HP string. This algorithm is based on a strategy of partitioning the entire protein sequence into two pieces. Our second algorithm partitions the HP string into four pieces and employs the idea of the first algorithm on the two halves. This gives a better approximation ratio of $\frac{5}{4}$ for *k *> 22. The latter result is applicable to HP strings where all the sequences of H's are of even length greater than two. The expected approximation ratio of this algorithm would be $\frac{5}{4}$ for *n *> 260 when both odd and even length sequences of H's having length greater than two are allowed. Here, *n *is the number of total H's in the HP string. We also present the idea of folding HP strings with sequences of H having length less than two. Notably, in the literature the best approximation ratio for the hexagonal lattice is 6, which is due to [9], and that for the square lattice with diagonals is $\frac{25}{16}$[6]. Clearly, the approximation ratio of our algorithm is better than the above results.

The rest of the paper is organized as follows. In Section 'Preliminaries', we introduce the hexagonal lattice with diagonals and define some related notions. Section 'Our Approaches' describes our algorithms and relevant results. We briefly conclude in Section 'Conclusion.

## Preliminaries

In this section, we present the required notions and notations to describe the hexagonal lattice model with diagonals.

**Definition **The two-dimensional hexagonal lattice with diagonals is an infinite graph *G *= (*V*, *E*) in Euclidian Space with vertex set *V *= ℝ^2 ^and edge set *E *= {(*x*, *x'*)*|x*, *x' *∈ ℝ^2^, *|x - x'| ≤ *2}, where *|.| *denotes the Euclidean norm. An edge *e ≡ *(*x*, *x'*) ∈ *E *is a non diagonal edge iff *|x - x'| *= 1; otherwise it is a diagonal edge.

We use the well known notion of neighbourhood or adjacency of graph theory: two vertices are adjacent/neighbour to each other if they are connected through an edge. In this connection, the difference between the usual hexagonal model and our propose model lies in the fact that a vertex in the former has three neighbours, whereas in the latter it has additional 9 neighbours, i.e., a total of twelve neighbours (see Figure 1).

Although the lattice is defined as an infinite graph, we will be concerned with only a finite sub-graph of it for each conformation of a protein. The input to the protein folding problem is a finite string *p *over the alphabet {*P*, *H*} where *p *= {*P*}*b_1_{*P*}^+^*b*_2_{*P*}^+^...{*P*}^+^*b_k_*{*P*}*. Here *b_i _*∈ {*H*}^+ ^*for *1 *≤ i ≤ k *and let $n=\sum_{i=1}^{k}|b_{i}|$. Here, H denotes non-polar and P denotes polar amino acids respectively. Often, in what follows, the input string in our problem will be refer to as an HP string. An H-run in an HP string denotes the consecutive H's and a P-run denotes consecutive P's. So, the total number of H-runs is *k *and total number of H is *n*. An H-run of even (odd) length is said to be an even H-run (odd H-run). We will now define the valid embeddings and conformation of a protein into this lattice. An embedding is a **self-avoiding walk **inside the grid.

**Figure 2:**
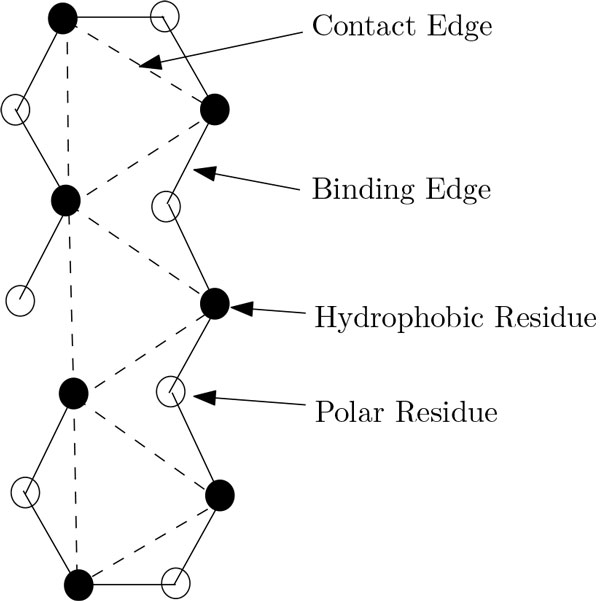
**Crossing between binding edges; this situation is forbidden in a valid conformation**. In this figure crossing between binding edges is illustrated. Notice that, this situation is forbidden in a valid conformation.

**Definition **Let *p *= *p*_1 _... *p_t _*be an HP string of length *t *and let *G *= (*V*, *E*) be a lattice. An embedding of *p *into *G *is a mapping function *f*: {1, ..., *t*} *→ V *from the positions of the string to the vertices of the lattice. It assigns adjacent positions in *p *to adjacent vertices in *G*, (*f*(*i*), *f*(*i *+ 1)) ∈ *E *for all 1 *≤ i ≤ t - *1. The edges (*f*(*i*), *f *(*i *+ 1)) ∈ *E *for 1 *≤ i ≤ t - *1 are called **binding edges**. An embedding of *p *into *G *is called a conformation, if no two binding edges cross each other (see Figure 2).

**Figure 3:**
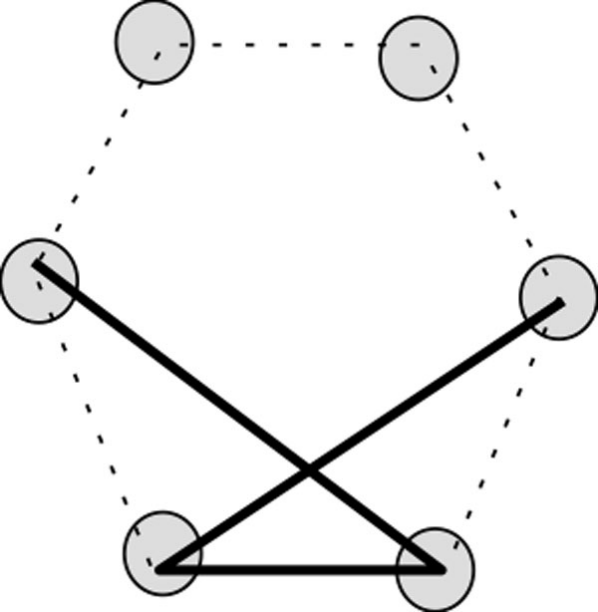
**A conformation of the string PHPHPHPHPHPHPH on the lattice**. In this figure, a conformation on hexagonal lattice with diagonal is illustrated.

In an conformation, a vertex occupied by an H (P) will often be referred to as an H-vertex (a P-vertex). Figure 3 shows an example of a conformation. Throughout the paper, H-vertices are indicated by filled circle and P-vertices are indicated by blank circles.

**Figure 4:**
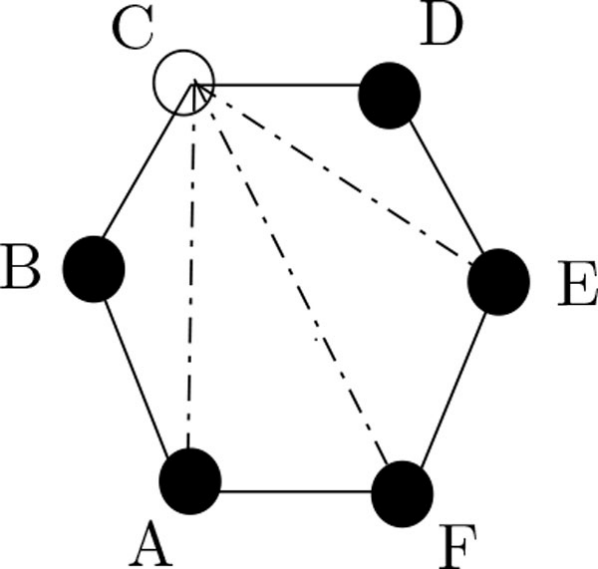
**(C, D) and (B, C) are alternating edges; (A, C), (C, F) and (C, E) are loss edges**. This figure aid to identify alternating edges and loss edges in a confirmation.

Definition Given a conformation *ϕ*, an edge (*x*, *x'*) of *G *is called a contact edge, if it is not a binding edge, but there exist *i*, *j *∈ {1, ..., *t*} such that *f*(*i*) = *x*, *f*(*j*) = *x'*, *and p_i _*= *p_j _*= *H*. The vertices of the lattice which are not occupied by an H or a P are called **unused vertices**. A binding edge connecting an H with a P is called an **alternating edge**. Loss edge is a non-binding edge incident to an H that is not a contact edge (see Figure 4).

Now, we define the neighbourhood of an edge in the lattice.

**Definition **Let *e *= (*x*, *y*) be any edge in *G*. We define the neighbourhood *N*(*e*) of *e *as the intersection of the neighbour of its endpoints *x *and *y*.

**Figure 5:**
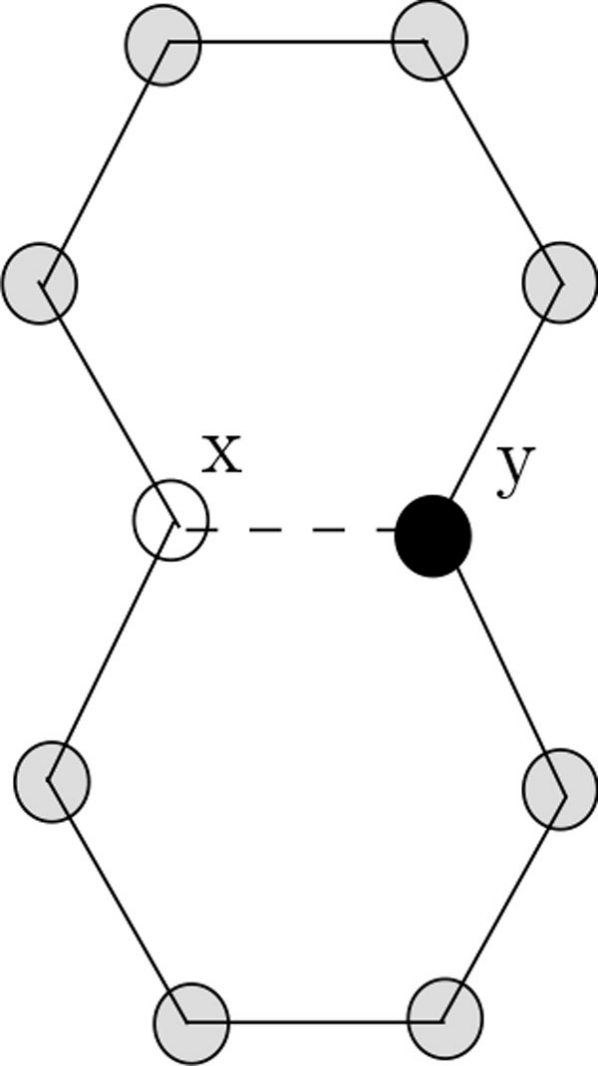
**Eight possible neighbours of the loss edge (*x*, *y*)**. This figure aids in understanding the proof of Lemma 0.2.

**Figure 6:**
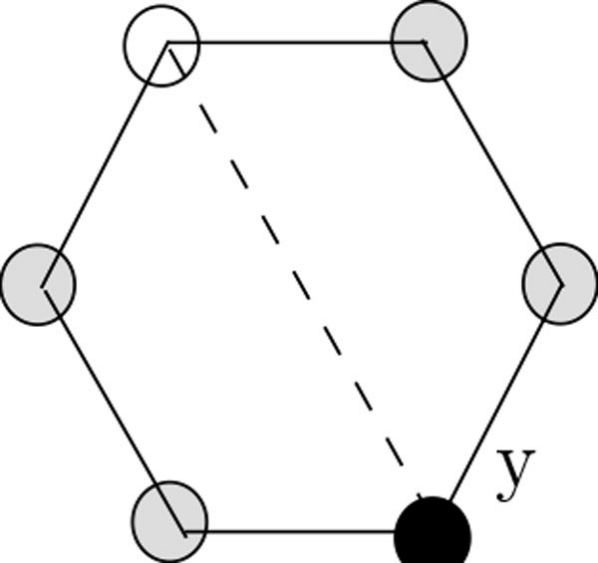
**Four possible neighbours of edge **(*x*, *y*) **when edge is diagonal**. This figure aids in understanding the proof of Lemma 0.2.

Neighbourhood of an edge *e *= (*x*, *y*) is shown in Figure 5 for non-diagonal edges, and in Figure 6 for diagonal edges. As can be seen from the figure for a non-diagonal edge, the number of possible neighbours is 8 whereas for a diagonal one, it is 4.

## Our approaches

In this section, we present two approximation algorithms for protein folding in a hexagonal lattice with diagonal. We start with deducing two upper bounds on the number of possible contacts for any H in the HP string.

### An upper bound

We will deduce a bound based on a simple counting argument: we will count the number of neighbours of a vertex in the lattice. We start with the following useful lemmas.

**Lemma 0.1 ***Let p be an HP string and G *= (*V*, *E*) *is a hexagonal lattice with diagonals. If p has a conformation in G*, *then any H in p can have at most ten contact edges*.

**Proof: **Every vertex in the lattice *G *has exactly twelve neighbours comprising 3 non-diagonal neighbours and 9 diagonal neighbours (see Figure 1). In this conformation, every H-vertex has exactly two binding edges. Hence 10 edges remain, which could potentially be contact edges. And hence the result follows. □

**Lemma 0.2 ***Let p be an input string for the problem and ϕ be a conformation of p. Let e = *(*x*, *y*) *be a loss edge with respect to ϕ. Then there are at most four alternating edges in N *(*e*).

**Proof: **If *e *is a non-diagonal edge, then the neighbourhood of *e *contains eight vertices (see Figure 5). If *e *is a diagonal edge, then the neighbourhood of *e *contains only four vertices (see Figure 6). Now, each of *x *and *y *can be incident to at most two binding edges. So, there are at most four binding edges in *N *(*e*). It follows immediately that there can be at most four alternating edges adjacent to *e*. □

Now we are ready to present the upper bound.

**Lemma 0.3 ***For a given HP string p*, *the the total number of contacts in a conformation ϕ is at most $\text{1}0n-\frac{\text{1}}{2}k$*, *where k is the total number of H-runs and n is the total number of H*.

**Proof : **From Lemma 0.1, we know that the number of contacts is at most 10*n*. In a confirmation one loss edge incident to H means that it would lose one contact edge. In what follows we will show that there will be at least $\frac{\text{1}}{2}k$ loss edges in *ϕ*. Since every H-run is preceded and followed by a total of two alternating edges, it is sufficient to prove that, for each alternating edge in *ϕ *for *p*, we have $\frac{\text{1}}{4}$ loss edge on average. From Lemma 0.2 we know that, for every loss edge there will be at most four alternating edges in its neighbourhood. Alternatively, we can say that, for every four alternating edges there will be at least one loss edge, assuming that the alternating edges are in the neighbourhood of that loss edge. Clearly, if the alternating edges are not within the neighbourhood then the number of loss edges will increase. So, for every alternating edge there will be at least $\frac{\text{1}}{4}$ loss edge. There are a total of 2*k *alternating edges. So, the total number of loss edges will be, $\frac{\text{1}}{4}\times \text{2}\times k=\frac{\text{1}}{2}k$. Hence, the result follows. □

### Algorithms and lower bounds

**Figure 7:**
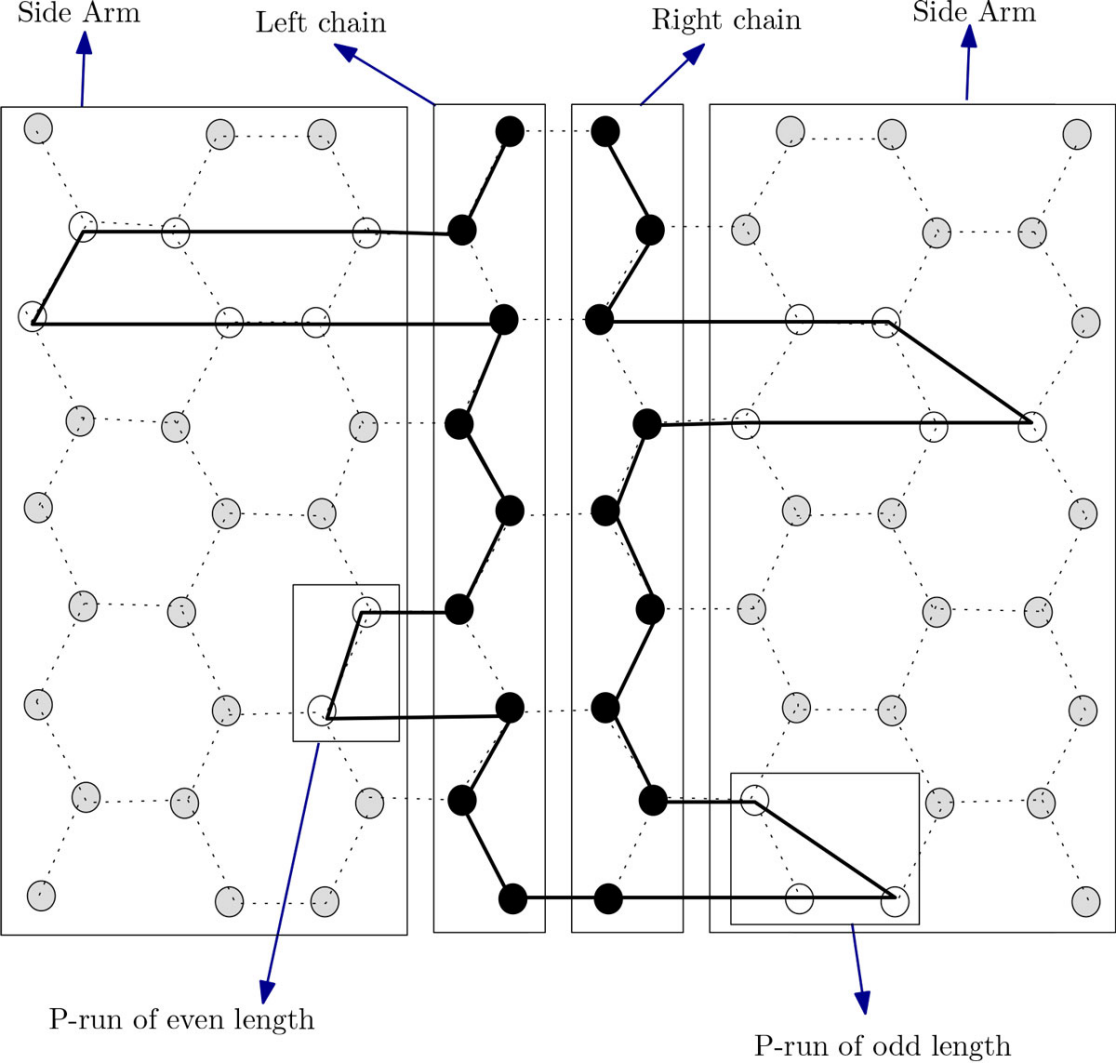
**Folding of HP string *H*^2^*P*^6^*H*^4^*P*^2^*H*^3^*P*^3^*H*^5^*P*^5^*H*^3 ^by Algorithm ChainArrangement**. This figure aids in understanding the folding by Algorithm ChainArrangement.

**Figure 8:**
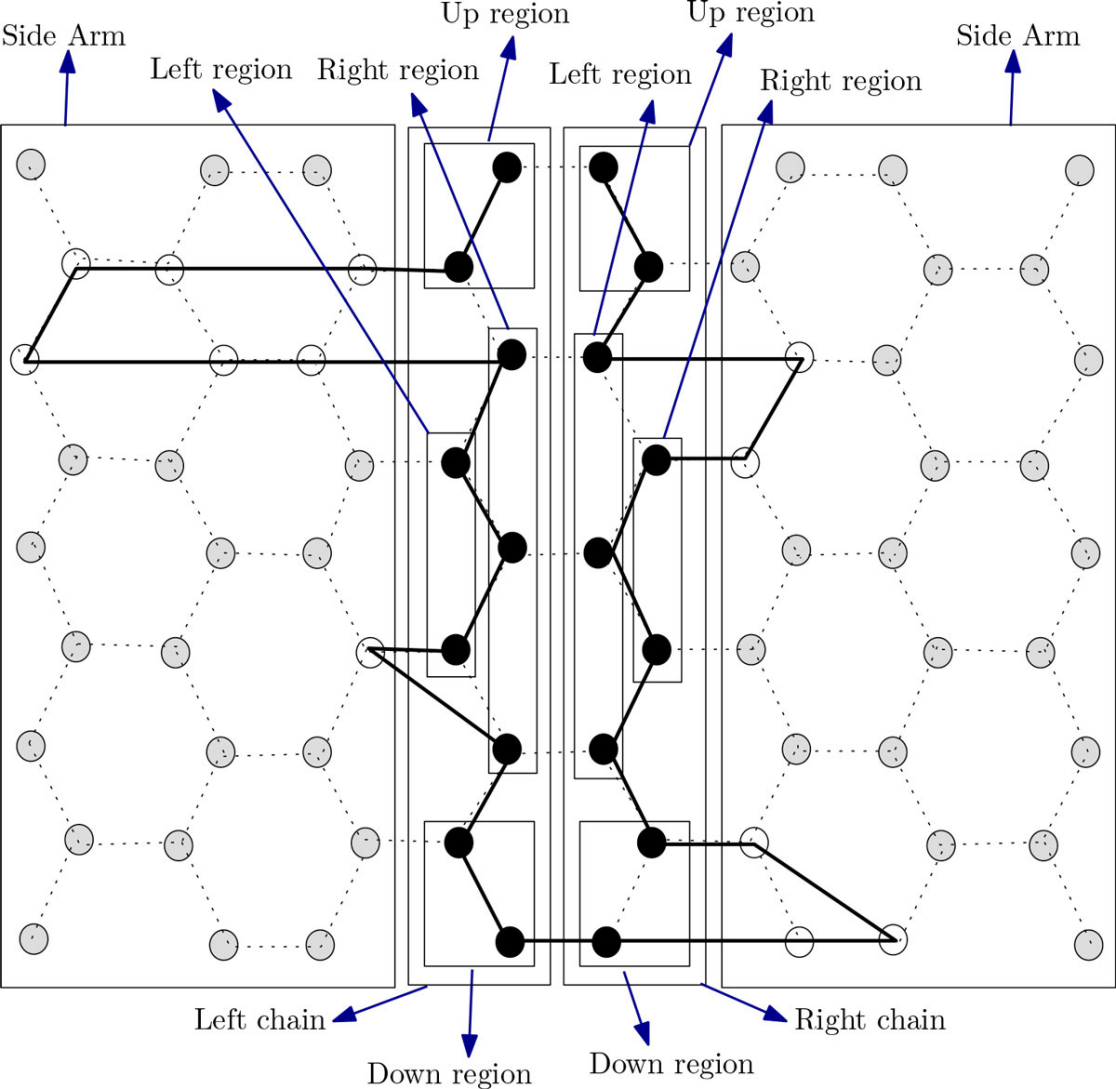
**Showing different regions of the left chain and the right chain for *m*_1 _= 2*x *+ 1**. This figure aids in finding the approximation ratio for Algorithm ChainArrangement.

**Figure 9:**
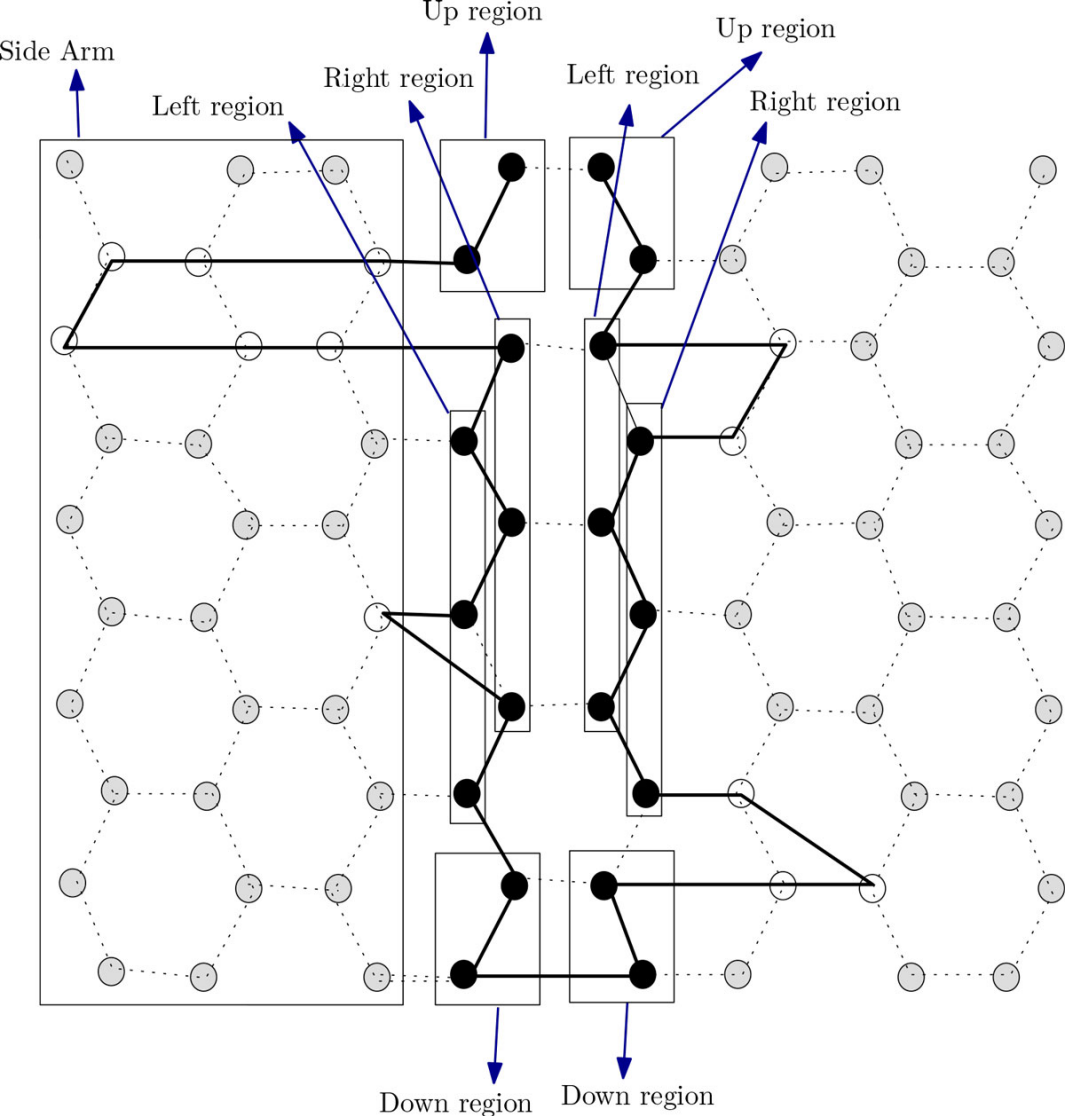
**Showing different portion of left chain and right chain for *m*_1 _= 2*x ***. This figure aids in finding the approximation ratio for Algorithm ChainArrangement.

In this section, we present two novel approximation algorithms for the problem. The idea of the first algorithm is to arrange all H's occurring in the input string along the two chains. We arrange the H's in the prefix of the string up to the $\left\lfloor \frac{n}{2}\right\rfloor$-th H on the left chain and arrange the rest of those on the right one (see Figure 7). Then we arrange the P's between H's outside these two chains. The arrangements of the P-runs along the side-arms of the two chains are shown in Figure 7. The arrangement in the left (right) chain can be further divided into four regions, namely, the left region, the right region, the up region and the down region (see Figure 8 and Figure 9). Now we formally present our algorithm in the form of Algorithm ChainArrangement.

Algorithm ChainArrangement

Input: An HP string *p*.

1. Set $f=\left\lfloor \frac{n}{2}\right\rfloor$.

2. Suppose *F *denotes the position in *p *after the *f*-th H. Denote by *pref F*(*p*) the prefix of *p *up to position *F *and by *suff F*(*p*) the suffix, that starts right after it. Now,

(a) Arrange the H's in *pref F*(*p*) along the left chain; intermediate P-runs are arranged in the side-arms of the left chain (see Figure 7).

(b) Arrange the H's in *suff F*(*p*) along the right chain; intermediate P-runs are arranged in the side-arms of the right chain (see Figure 7).

### Approximation ratio for Algorithm ChainArrangement

Now we focus on deducing an approximation ratio for Algorithm ChainArrangement. Suppose that $m_{1}=\left\lfloor \frac{n}{2}\right\rfloor$. So, according to Algorithm ChainArrangement, the left (right) chain will contain *m*_1 _(*m*_1 _or *m*_1 _+ 1) H's. We need to consider two cases, namely, where *m*_1 _= 2*x *+ 1 and *m*_1 _= 2*x*, with an integer *x *> 0. In what follows, we will use *vw*-left chain (*vw*-right chain) to denote a particular region of the left (right) chain. So, *vw *could be one of the 4 options, namely, *lR *(left region), *rR *(right region), *uR *(up region) and *dR *(down region). We also use *ϕ_CA _*to refer to the conformation given by Algorithm ChainArrangement.

***case 1: ****m*_1 _= 2*x *+ 1

The analysis for this case will be easier to understand with the help of Figure 8. Suppose *n *is even. In *ϕ_CA_*, every vertex in the *lR*-left chain has at least 5 contacts. There are a total of *x *- 2 such vertices (see Figure 8 and Table 1). Every vertex in the *rR*-left chain has at least 7 contacts. There are a total of *x *- 1 such vertices. The two vertices in the *uR*-left chain each has at least 4 contacts. One vertex in the *dR*-left chain has at least 4 contacts while the other has at least 3 contacts.

**Table 1:** Number of vertices in each region in left chain *ϕ*_*CA*_

Region	*m*_1 _= 2*x *+ 1	*m*_1 _= 2*x*
*lR*-left chain	*x *- 2	*x *- 2

*rR*-left chain	*x *- 1	*x *- 2

*uR*-left chain	2	2

*dR*-left chain	2	2

This table illustrates the number of vertices in each region in left chain *ϕ*_*CA*_.

So, the total number of contacts (C) of all the vertices in the left chain, can be computed as follows:

$$\begin{array}{c} C\geq \text{5}\times \left(x-\text{2}\right)+\text{7}\times \left(x-\text{1}\right)+\text{4}\times \text{3}+\text{3}\\ \Rightarrow C\geq \text{5}x-\text{1}0+\text{7}x-\text{7}+\text{15}\\ \Rightarrow C\geq \text{12}x-\text{2}\\ \Rightarrow C\geq \text{12}x+\text{6}-\text{8}\\ \Rightarrow C\geq \text{6}\left(\text{2}x+\text{1}\right)-\text{8}\\ \Rightarrow C\geq \text{6}m_{\text{1}}-\text{8}\\ \Rightarrow C\geq \text{3}n-\text{8} \end{array}$$

Since the right chain is symmetric to the left one, both chains will have the same number of vertices if *n *= 2*m*_1_, i.e., all the vertices of the right chain will also have at least  C contacts. So the total number of contacts will be at least 2C or 6*n *- 16.

If *n *= 2*m*_1 _+ 1 then let *n*_1 _= *n *- 1. This *n*_1 _vertices will have at least 6*n*_1 _- 16 contacts. The remaining vertex will have at least 2 contacts. So the total number of contacts will be at least 6(*n *- 1) - 16 + 2 or 6*n *- 20.

***case 2: **m*_1 _= 2*x*

The analysis of this case will be easy to understand with the help of Figure 9. Let *n *is even. In *ϕ_CA_*, every vertex in the *lR*-left chain has at least 5 contacts. There are a total of *x *- 2 such vertices (see Figure 9). Every vertex in the *rR*-left chain has at least 7 contacts. There are a total of *x *- 2 such vertices. The two vertices in the *uR*-left chain each has at least 4 contacts. One vertex in the *dR*-left chain has at least 5 contacts while the other has at least 2 contacts.

So, the total number of contacts (C) of all the vertices of the left chain can be computed as follows:

$$\begin{array}{c} C\geq \text{5}\times \left(x-\text{2}\right)+\text{7}\times \left(x-\text{2}\right)+\text{4}\times \text{2}+\text{5}+\text{2}\\ \Rightarrow C\geq \text{5}x-\text{1}0+\text{7}x-\text{14}+\text{15}\\ \Rightarrow C\geq \text{12}x-\text{9}\\ \Rightarrow C\geq \text{6}m_{\text{1}}-\text{9}\\ \Rightarrow C\geq \text{3}n\;-\text{9} \end{array}$$

Since the right chain is symmetric to the left one, both chains will have the same number of vertices if *n *= 2*m*_1_. So all the vertices of the right chain will also have at least  C contacts. So the total number of contacts will be at least 2C or 6*n *- 18.

If *n *= 2*m*_1 _+ 1 and *m*_1 _= 2*x *then let *n*_1 _= *n *- 1. This *n*_1 _vertices will have at least 6*n*_1 _- 18 contacts. The remaining vertex will have at least 2 contacts. So the total number of contacts will be at least 6(*n - *1) *-*18 + 2 or 6*n *- 22.

So, combining the two cases, we get that the total number of contacts is at least 6*n *- 22. Now we need to take the alternating edges into our consideration. For every alternating edge we get two extra contacts for the two vertices (each having one). So, for *n *H's and *k *alternating edges we get a total of at least 6*n *- 22 + 2*k *contacts. Hence we get the following approximation ratio *A*_1_:

(1) $$A_{1}=\frac{10n-\frac{1}{2}k}{\left(6n-22+2k\right)}$$

From Equation 1 it can be seen that for large *n*, *A*_1 _tends to reach $\frac{10}{6}$. So we compute the value of *k *so that our approximation ratio is at most $\frac{10}{6}$ as shown below.

$$\begin{array}{l} \;\frac{10n-\frac{k}{2}}{\left(6n-22+2k\right)}\leq \frac{10}{6}\\ \;\Rightarrow 10n-\frac{k}{2}\leq \frac{10}{6}\times (6n-22+2k)\\ \;\Rightarrow 10n-\frac{k}{2}\leq 10n-\frac{110}{3}+\frac{10k}{3})\\ \;\Rightarrow \frac{10k}{3}+\frac{k}{2}\geq \frac{110}{3}\\ \;\Rightarrow \frac{23k}{6}\geq \frac{110}{3}\\ \;\Rightarrow k\geq \frac{220}{23}\approx 9.6 \end{array}$$

So, if the total number of H-runs is greater than 9, then Algorithm ChainArrangement will achieve an approximation ratio of $\frac{10}{6}$ or $\frac{5}{3}$.

Note that, the value of *k *is dependent on *n *and the HP string. We now deduce the expected value of *k *for a given HP string. This problem can be mapped into the problem of *Integer Partitioning *as defined below. Notably, similar mapping has recently been utilized in [16] for deriving an expected approximation ratio of another algorithm.

**Problem 0.4 ***Given an integer Y *, *the problem of Integer Partitioning aims to provide all possible ways of writing Y *, *as a sum of positive integers*.

Note that the ways that differ only in the order of their summands are considered to be the same partition. A summand in a partition is called a part. Now, if we consider *n *as the input of Problem 0.4 (i.e., *Y*) then each length of H-runs can be viewed as parts of the partition. So if we can find the expected number of partitions we could in turn get the expected value of *k*. Kessler and Livingston [17] showed that to get an integer partition of an integer *Y *, expected number of required parts is:

$$\sqrt{\frac{3Y}{2\pi }}\times \left(\text{log}Y\;+\;2\gamma \;-\;2\text{log}\sqrt{\frac{\pi }{6}}\right),$$

where *γ *is the famous Euler's constant.

For our problem *Y *= *n*. If we denote *E*[*P *] as the expected number of H-runs then,

$$E\left[P\right]=\sqrt{\frac{6}{\pi }}\times \sqrt{n}\times \left(\frac{1}{2}\text{log}n+\gamma -\text{log}\sqrt{\frac{\pi }{6}}\right).$$

Now, as $\left(\frac{1}{2}\text{log}\;n+\gamma -\text{log}\sqrt{\frac{\pi }{6}}\right)\leq \left(\sqrt{\frac{2\pi }{3}}\times \frac{\text{1}}{2}\text{log}\;n\right)$ for *n ≥ *5, we can say that

$$E\left[P\right]\leq \sqrt{n}\times \text{log}n.$$

So the expected value of *k *is less than or equal to $\sqrt{n}\times \text{log}\;n$ which implies that $\sqrt{n}\times \text{log}n\geq \frac{220}{3}$ or *n ≥ *16. The above findings are summarized in the form of the following theorems.

**Theorem 0.5 ***For any given HP string*, *Algorithm ChainArrangement gives a *$\frac{5}{3}$*approximation ratio for k *> 10, *where k is the total number of H-runs*. □

**Theorem 0.6 ***For any given HP string*, *Algorithm ChainArrangement is expected to achieve an approximation ratio of *$\frac{5}{3}$*for n ≥ *16, *where n is the total number of H*. □

### An improved algorithm

**Figure 10:**
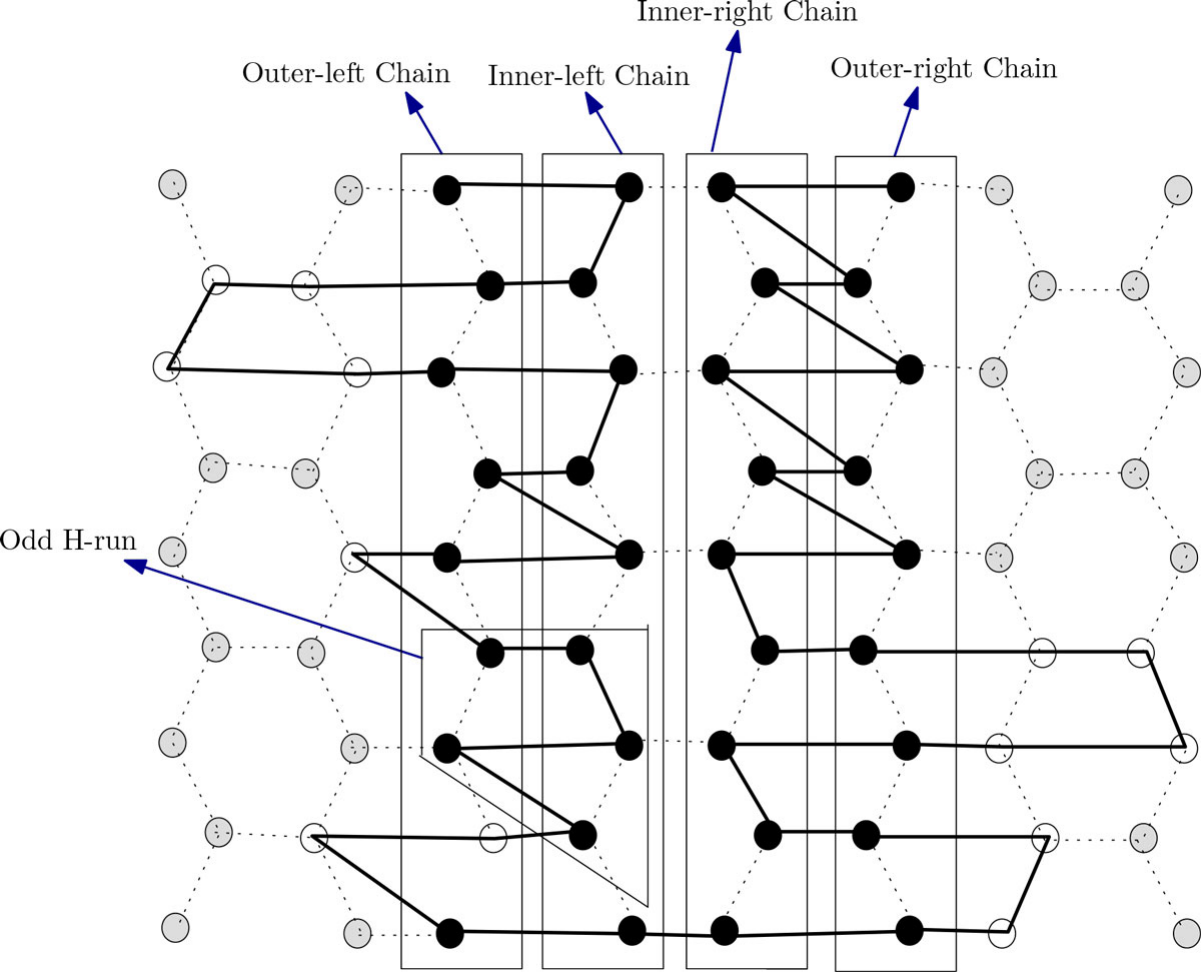
**Folding of HP string *H*^4^*P*^4^*H*^6^*PH*^5^*P*^2^*H*^4^*P*^2^*H*^4^*P*^4^*H*^1^^2 ^by the Algorith ImprovedChainArrangement**. This figure aids in understanding the folding by Algorithm ImprovedChainArrangement.

**Figure 11:**
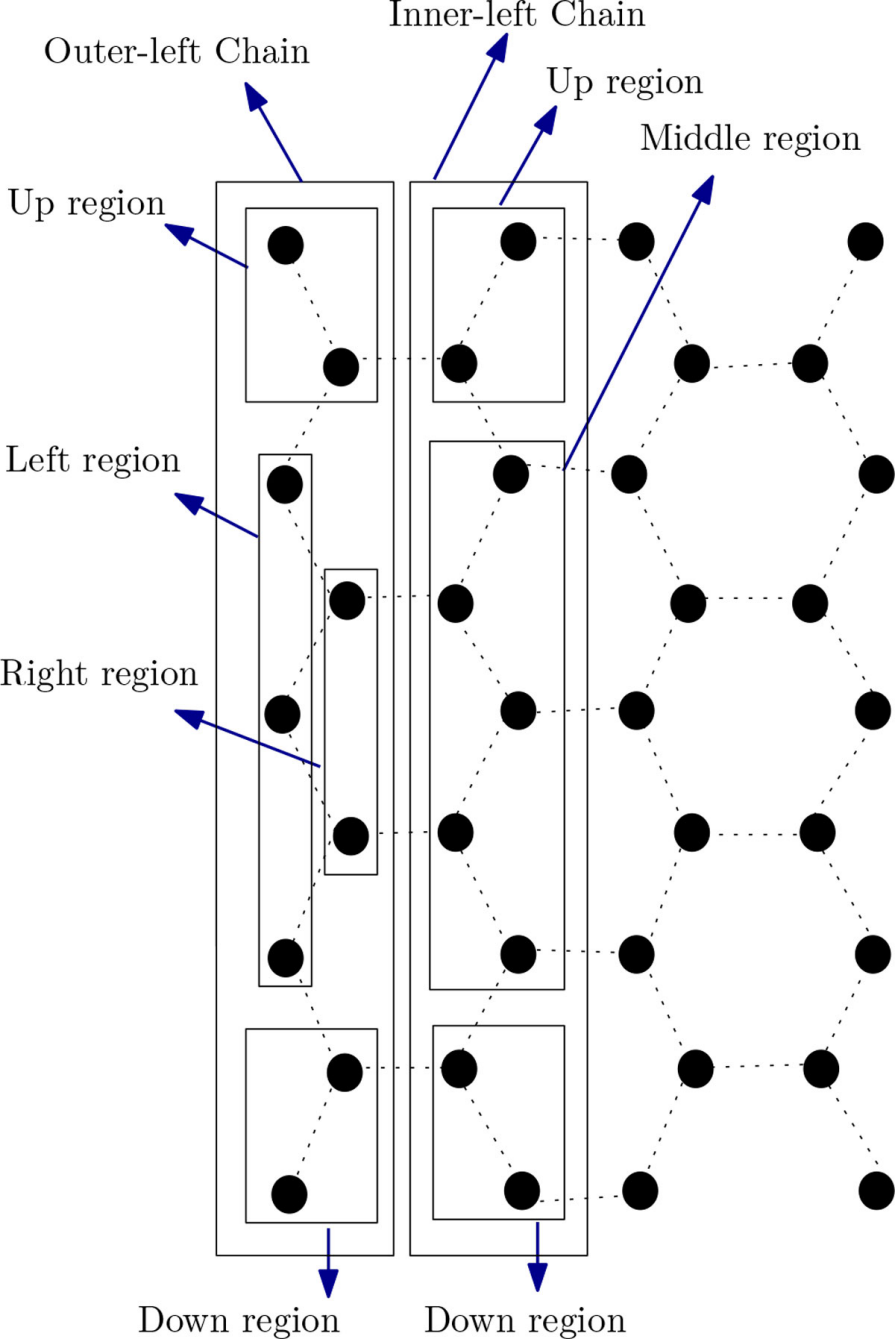
**Showing diiferent region of Inner-left-chain and Outer-left-chain**. This figure aids in finding the approximation ratio for Algorithm ImprovedChainArrangement.

From Equation 1 we can see that a higher value of *k *will give us a better approximation ratio. So, if there are many short H-runs then we will get better results. This interesting insight provides us with an idea of an improved algorithm. However, as will be discussed later, the better approximation ratio will be applicable only if every H-run is of length greater than 2. For this improved algorithm we introduce the notion of inner-left chains, outer-left chains, inner-right chains and outer-right chains as shown in Figure 10. Recall that, unlike the current algorithm, there were only two chains in our previous algorithm. The arrangement in the outer-left (outer-right) chain can be further divided into four regions, namely, the left region, the right region, the up region and the down region. The arrangement in the inner-left (inner-right) chain can be further divided into three regions, namely, the middle region, the up region and the down region (see Figure 11). We apply the following procedures. We first put an H of an H-run in the outer-left chain; the next two H's of the H-run is placed in the inner-left chain. Rest of the H's of the H-run are placed alternatively on the inner-left chain (inner-right chain) and on the outer-left chain (outer-right chain) (see Figure 10). At this point, a brief discussion on the difference between the arrangements done by the two algorithm is in order. In Algorithm ChainArrangement, we can place all P's of an HP string in the side arms. However in the current algorithm we may have to arrange some P's of an HP string in the outer-left chain or outer-right chain also (see Figure 10). The algorithm is finally presented below.

### Algorithm ImprovedChainArrangement

Input: An HP string *p *such that every H-run is of length greater than two.

1. Set $f=\left\lfloor \frac{n+k_{1}}{4}\right\rfloor$, where *k*_1 _denotes total number of odd H-runs.

2. Suppose *F *denotes the position in *p *after the *f*-th H. Denote by *pref F*(*p*) the prefix of *p *up to position *F *and by *suff F*(*p*) the suffix, that starts right after it. Now, place the H-runs of *pref F*(*p*) in the outer-left chain and the inner-left chain as follows.

(a) First put an H of an H-run in the outer-left chain; then put the next two H's of it in the inner-left chain.

(b) Arrange the rest of the H's alternatively, between the outer-left chain and the inner-left chain.

(c) If the current H-run ends at the outer-left chain, the P-run following it is placed in the side-arms of the outer-left chain; otherwise, the H-run ends at the inner-left chain (i.e., odd H-runs), and hence the first P of the P-run following it is placed at the outer-left chain. Finally the rest of the P's of the P-run are arranged in the side-arms of the outer-left chain (see Figure 10).

And place the H-runs of *suff F*(*p*) in the outer-right chain and the inner-right chain as follows.

(a) First put an H of an H-run in the outer-right chain; then put the next two H's of it in the inner-right chain.

(b) Arrange the rest of the H's alternatively, between the outer-right chain and the inner-right chain.

(c) If the current H-run ends at the outer-right chain, the P-run following it is placed in the side-arms of the outer-right chain; otherwise the H-run ends at the inner-right chain (i.e. odd H-runs), and hence the first P of the P-run following it is placed at outer-right chain. Finally the rest of the P's of the P-run are arranged in the side-arms of outer-right chain (see Figure 10).

### Approximation ratio for Algorithm ImprovedChainArrangement

In this section, we deduce the approximation ratio for Algorithm ImprovedChainArrangement. We present our analysis in two separate cases. In Case 1, we only have even H-runs in HP strings. In Case 2 we may also have odd H-runs in HP strings. In what follows, we will use *vw*-outer-left chain (*vw*-outer-right chain) to denote a particular region of the outer-left (outer-right) chain. So, *vw *could be one of the 4 options, namely, *lR *(left region), *rR *(right region), *uR *(up region) and *dR *(down region). We also use *vw*-inner-left chain (*vw*-inner-right chain) to denote a particular region of the inner-left (inner-right) chain. So, *vw *could be one of the 3 options, namely, *mR *(middle region), *uR *(up region) and *dR *(down region). Furthermore we use *ϕ_ICA _*to refer to the conformation given by Algorithm ImprovedChainArrangement.

#### HP string contains only even H-runs

Suppose that all the H-runs are of even length. Let *n *= 4*m*_2 _and *m*_2 _= 2*x *+ 1, where *x *> 0 is an integer. In *ϕ_ICA _*every vertex in the *mR*-inner-left chain has at least 10 contacts (see Figure 11 and Table 2). There are a total of 2*x *- 3 such vertices. One vertex in the *uR*-inner-left chain and one vertex in the *dR*-inner-left chain has at least 4 contacts each. The other vertices in the *uR*-inner-left chain and *dR*-inner-left chain has at least 7 contacts each.

**Table 2:** Number of vertices in each region in inner-left chain and outer-left chain *ϕ*_*ICA*_

Region	Outer-left chain	Inner-left chain
Left region	*x *- 1	N/A

Right region	*x *- 2	N/A

Middle region	N/A	2*x *- 3

Up region	2	2

Down region	2	2

This table illustrates the number of vertices in each region in inner-left chain and outer-left chain *ϕ*_*ICA*_.

So, the total number of contacts of all the vertices of the inner-left chain, $C_{1}$ can be computed as follows:

$$\begin{array}{c} C_{\text{1}}\geq \text{1}0\times \left(\text{2}x-\text{3}\right)+\text{2}\times \text{4}+\text{2}\times \text{7}\\ \Rightarrow C_{\text{1}}\geq \text{2}0x-\text{8}\\ \Rightarrow C_{\text{1}}\geq \text{2}0x+\text{1}0-\text{18}\\ \Rightarrow C_{\text{1}}\geq \text{1}0\left(\text{2}x+\text{1}\right)-\text{18}\\ \Rightarrow C_{\text{1}}\geq \text{1}0m_{\text{2}}-\text{18} \end{array}$$

Since the inner-left chain and the inner-right chain are symmetric to each other, all the vertices of the inner-right chain will also have at least $C_{1}$ contacts. So the total number of contacts in the inner-left chain and the inner-right chain will be at least $\text{2}C_{1}$ or 20*m*_2 _- 36.

Now, let us consider the outer-left chain and outer-right chain. Every vertex in the *lR*-outer-left chain has at least 5 contacts (see Figure 11 and Table 2). There are a total of *x *- 1 such vertices. Every vertex in the *rR*-outer-left chain has at least 7 contacts. There are a total of *x *- 2 such vertices. One vertex in the *uR*-outer-left chain and one vertex in the *dR*-outer-left chain has at least 4 contacts each. Each of the other vertices in the *uR*-outer-left chain and *dR*-outer-left chain has at least 5 contacts.

So, the total number of contacts of all the vertices of outer-left chain, $C_{2}$ can be computed as follows:

$$\begin{array}{c} C_{\text{2}}\geq \text{5}\times \left(x-\text{1}\right)+\text{7}\times \left(x-\text{2}\right)+\text{2}\times \text{4}+\text{2}\times \text{5}\\ \Rightarrow C_{\text{2}}\geq \text{5}x-\text{5}+\text{7}x-\text{14}+\text{18}\\ \Rightarrow C_{\text{2}}\geq \text{12}x-\text{1}\\ \Rightarrow C_{\text{2}}\geq \text{12}x+\text{6}-\text{7}\\ \Rightarrow C_{\text{2}}\geq \text{6}\left(\text{2}x+\text{1}\right)-\text{7}\\ \Rightarrow C_{\text{2}}\geq \text{6}m_{\text{2}}-\text{7} \end{array}$$

Since the outer-left chain and the outer-right chain are symmetric to each other, all the vertices of the outer-right chain will also have at least $C_{2}$ contacts. So total number of contacts in the outer-left chain and the outer-right chain will be at least $\text{2}C_{2}$ or 12*m*_2 _- 14. So, the number of total contacts will be at least 20*m*_2 _- 36 + 12*m*_2 _- 14 = 32*m*_2 _- 50 = 8*n *- 50.

So far we have assumed *n *= 4*m*_2 _and *m*_2 _= 2*x *+ 1. Now we consider the case where *n *= 4*m*_2 _and *m*_2 _= 2*x *such that *x *> 0 is an integer. For this case, we can do a similar analysis to compute the total number of contacts, which will be the same, i.e., 8*n *- 50. So when all the H-runs have even length, we get that the total number of contacts is at least 8*n *- 50. Now we consider the alternating edges. For every alternating edge we get two extra contacts for the two corresponding vertices (each having one). So, for *n *number of H's and *k *alternating edges we get a total of at least 8*n *- 50 + 2*k *contacts. Hence we get the following approximation ratio *A*_2_,

(2) $$A_{2}=\frac{10n-\frac{1}{2}k}{\left(8n-50+2k\right)}$$

From Equation 2 it can be seen that for large *n*, *A*_2 _tends to reach $\frac{10}{8}$. So we are going to find the value of *k *for which our approximation ratio will be at most $\frac{10}{8}$ or $\frac{5}{4}$.

$$\begin{array}{l} \;\frac{10n-\frac{k}{2}}{\left(8n-50+2k\right)}\leq \frac{10}{8}\\ \;\Rightarrow 10n-\frac{k}{2}\leq \frac{10}{8}\times (8n-50+2k)\\ \;\Rightarrow 10n-\frac{k}{2}\leq 10n-\frac{125}{2}+\frac{5k}{2})\\ \;\Rightarrow \frac{5k}{2}+\frac{k}{2}\geq \frac{125}{2}\\ \;\Rightarrow \frac{6k}{2}\geq \frac{125}{2}\\ \;\Rightarrow k\geq \frac{125}{6}\approx 20 \end{array}$$

Note that, the value of *k *is dependent on *n *and the HP string. We now deduce the expected value of *k *for a given HP string such that each H-run is even and has length greater than two. Again, this problem can be mapped into the problem of *Integer Partitioning*, and hence, as before, the expected value of *k *is less than or equal to $\sqrt{n}\times \text{log}\;n$ which implies $\sqrt{n}\times \text{log}\;n\geq \frac{125}{6}\;\text{or}\;n\geq 22$. The above results can be summarized in the form of following theorems.

**Theorem 0.7 ***For any given HP string such that each H-run is even and has length greater than two*, *Algorithm ImprovedChainArrangement achieves an approximation ratio of *$\frac{5}{4}$*for k *> 20 *where k is the total number of H-runs*. □

**Theorem 0.8 ***For any given HP string such that each H-run is even and has length greater than two*, *it is expected that Algorithm ImprovedChainArrangement would achieve an approximation ratio of *$\frac{5}{4}$*for n ≥ *22 *where n is the total number of H's*. □

#### HP string contains both odd H-runs and even H-runs

So far we have assumed that the given HP string contains only even H-runs. Now we are going to consider the case where both odd and even H-runs are present. Let *k*_1 _is the total number of odd H-runs. According to the Steps 3 and 4 of Algorithm ImprovedChainArrangement, we have to put P in the outer-left chain or outer-right chain for each odd H-run. So, the total number of P's in the outer-left chain or the outer-right chain is *k*_1_. Let, *n*_2 _= *n *+ *k*_1_. We will loose at most 14 (10) contacts for each P in the left (right) region of the outer-left chain and same will happen for the outer-right chain. So, on an average, we lose 12*k*_1 _contacts for such placement of P due to odd H-runs. So, from Equation 2, we get the following expected approximation ratio *A*_3_,

$$\begin{array}{c} \;\;\;\;A_{3}=\frac{10n-\frac{1}{2}k}{\left(8n_{2}-50+2k-12k_{1}\right)}\\ \Rightarrow A_{3}=\frac{10n-\frac{1}{2}k}{\left(8n+8k_{1}-50+2k-12k_{1}\right)}\\ \Rightarrow A_{3}=\frac{10n-\frac{1}{2}k}{\left(8n-50+2k-4k_{1}\right)} \end{array}$$

Assuming that an H-run can be odd or even with equal probability, we get *k *= 2*k*_1_. Then we can simplify as follows: $A_{3}=\frac{10n-\frac{1}{2}k}{\left(8n-50+2k-2k\right)}=\frac{10n-\frac{1}{2}k}{\left(8n-50\right)}.$

This gives us an approximation ratio for the case when H-runs could be both odd and/or even under the assumption that H-run could be odd or even with equal probability. Now we are going to find the value of *k *so that our expected approximation ratio will be at most $\frac{10}{8}$ or $\frac{5}{4}$.

$$\begin{array}{c} \;\;\;\;\frac{10n-\frac{k}{2}}{\left(8n-50\right)}\leq \frac{10}{8}\\ \;\Rightarrow 10n-\frac{k}{2}\leq \frac{10}{8}\times \left(8n-50\right)\\ \;\Rightarrow 10n-\frac{k}{2}\leq 10n-\frac{500}{8}\\ \;\Rightarrow \frac{k}{2}\geq \frac{500}{8}\\ \;\Rightarrow k\geq \frac{1000}{8}=125 \end{array}$$

Note that, the value of *k *is dependent on *n*. To get an idea on the expected behaviour of our algorithm, we now deduce the expected value of *k *for a given HP string such that H-runs can be even or odd and has length greater than two. Again, this problem can be mapped into the problem of *Integer Partitioning*, So the expected value of *k *is less than or equal to $\sqrt{n}\times \text{log}\;n$ which implies $\sqrt{n}\times \text{log}\;n\geq 125\;\text{or}\;n\geq 260$. The results discussed above can be summarised in the form of following theorem.

**Theorem 0.9 ***For any given HP string such that H-runs can be even or odd and has length greater than two*, *it is expected that Algorithm ImproveChainArrangement gives a *$\frac{5}{4}$*approximation ratio for n ≥ *260.

#### H-runs of length 1 and 2

**Figure 12:**
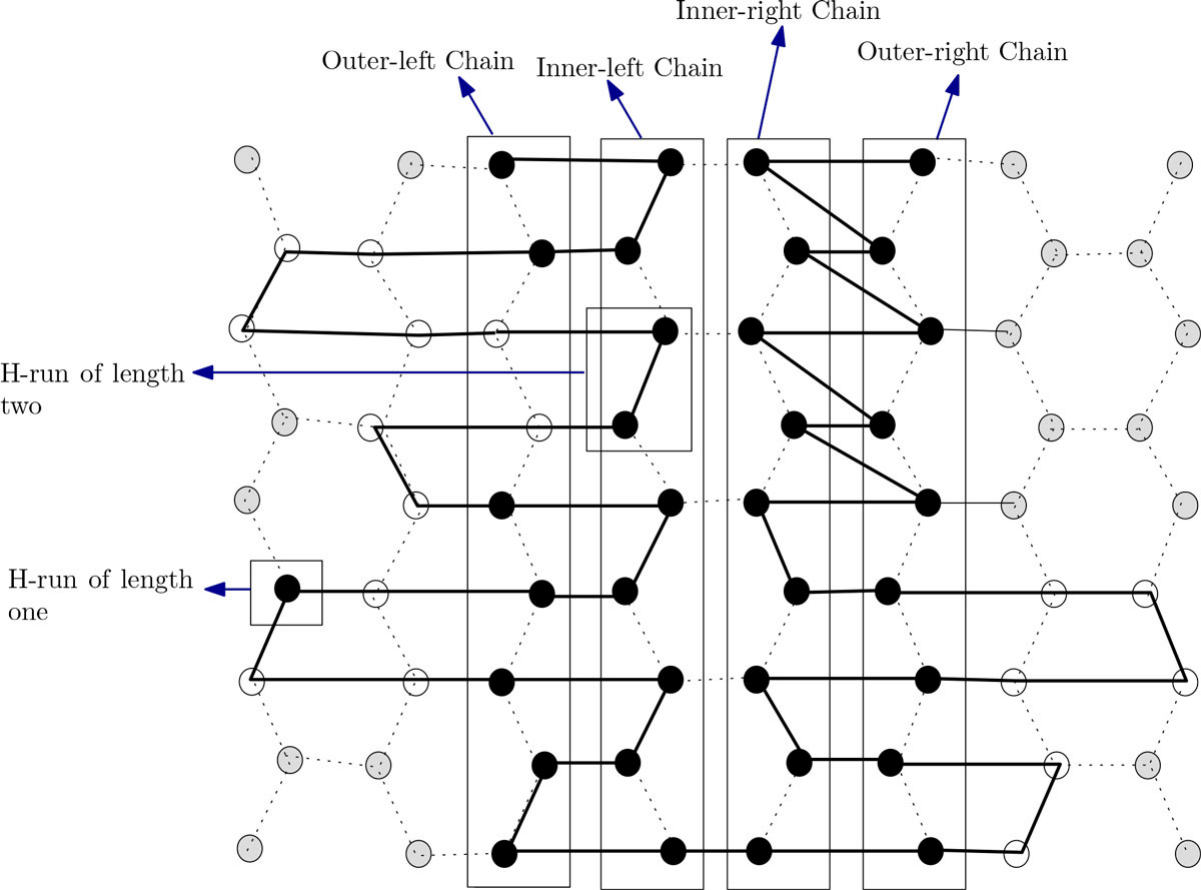
**Folding of H-runs having length one and two**. This figure aids in finding the approximation ratio for Algorithm ImprovedChainArrangement.

Although in our analysis we excluded the HP-string having H-runs with length less than 3, below we discuss, how we can arrange such H-runs to get a folding using our approach. We can arrange HP-strings with H-runs of length 2 in the inner-left chain (inner-right chain) as shown in Figure 12. For each H-run of length two we will lose 24 contacts. If the total number of such H-runs is *k*_2_, then we will lose at most 24*k*_2 _contacts. If we have HP strings having H-runs of length one, we can arrange this at the outside of the outer-left chain (outer-right chain) as shown in Figure 12. For each H-run of length one we will lose 20 contacts. If the total number of such H-runs is *k*_3_, then we will lose at most 20*k*_3 _contacts.

## Conclusion

In this paper, we have introduced hexagonal lattice with diagonals for the protein folding problem in the HP model. We have presented two novel approximation algorithms for protein folding in this lattice. Our first algorithm is a $\frac{5}{3}$-approximation algorithm for *k *> 10 where *k *is the number of H-runs in the HP string. Our second algorithm gives a better approximation ratio of $\frac{5}{4}$ for *k *> 22. The latter result is applicable to HP strings where the H-runs are of even length greater than two. The expected approximation ratio of this algorithm would be $\frac{5}{4}$ for *n *> 260 when both odd and even length H-runs having length greater than two are allowed (*n *is the number of total H's in the HP string). Notably the best approximation ratio for hexagonal lattice is 6, which is due to [9], and the approximation ratio for square lattice with diagonal is $\frac{25}{16}$[6]. Clearly the approximation ratio of our algorithm is better than the above result.

## Competing interests

The authors declare that they have no competing interests.

## Authors' contributions

Both Shaw and Islam perceived the study, proposed the models and the algorithms. Both of them along with Rahman conducted and verified the analysis. The total work was supervised by Rahman and Hasan.

All authors wrote and approved the manuscript.

## Declaration

The publication costs for this article were partially funded by a small grant from BRAC University, Bangladesh and a CodeCrafters-Investortools Research Grant. A significant part of the costs have been borne by the authors.

This article has been published as part of *BMC Bioinformatics *Volume 15 Supplement 2, 2014: Selected articles from the Twelfth Asia Pacific Bioinformatics Conference (APBC 2014): Bioinformatics. The full contents of the supplement are available online at http://www.biomedcentral.com/bmcbioinformatics/supplements/15/S2.
